# The magnitude and characteristics of the population of cancer survivors: using population-based estimates of cancer prevalence to inform service planning for survivorship care

**DOI:** 10.1186/1471-2407-14-767

**Published:** 2014-10-15

**Authors:** Linda Sharp, Sandra Deady, Pamela Gallagher, Michal Molcho, Alison Pearce, Audrey Alforque Thomas, Aileen Timmons, Harry Comber

**Affiliations:** National Cancer Registry, Building 6800, Cork Airport Business Park, Kinsale Road, Cork, Ireland; Dublin City University, Dublin, Ireland; National University of Ireland, Galway, Ireland

**Keywords:** Cancer, Prevalence, Survivors, Supportive care, Unmet needs

## Abstract

**Background:**

Rising cancer incidence and survival mean that the number of cancer survivors is growing. Accumulating evidence suggests many survivors have long-term medical and supportive care needs, and that these needs vary by survivors’ socio-demographic and clinical characteristics. To illustrate how cancer registry data may be useful in survivorship care service planning, we generated population-based estimates of cancer prevalence in Ireland and described socio-demographic and clinical characteristics of the survivor population.

**Methods:**

Details of people diagnosed with invasive cancer (ICD10 C00-C96) during 1994–2011, and who were still alive on 31/12/2011, were abstracted from the National Cancer Registry, and tabulated by cancer site, sex, current age, marital status, initial treatment, and time since diagnosis. Associations were investigated using chi-square tests.

**Results:**

After excluding non-melanoma skin cancers, 17-year cancer prevalence in Ireland was 112,610 (females: 58,054 (52%) males: 54,556 (48%)). The four most prevalent cancers among females were breast (26,066), colorectum (6,598), melanoma (4,593) and uterus (3,505) and among males were prostate (23,966), colorectum (8,207), lymphoma (3,236) and melanoma (2,774). At the end of 2011, 39% of female survivors were aged <60 and 35% were ≥70 compared to 25% and 46% of males (p < 0.001). More than half of survivors of bladder, colorectal and prostate cancer were ≥70. Cancers with the highest percentages of younger (<40) survivors were: testis (50%); leukaemia (females: 28%; males: 22%); cervix (20%); and lymphoma (females: 19%; males: 20%). Fewer female (57%) than male (64%) survivors were married but the percentage single was similar (17-18%). More female (25%) than male survivors (18%; p < 0.001) were ≥10 years from diagnosis. Overall, 69% of survivors had undergone cancer-directed surgery, and 39%, 32% and 18% had received radiotherapy, chemotherapy and hormone therapy, respectively. These frequencies were higher among females than males (surgery: 82%, 54%; radiotherapy: 42%, 35%; chemotherapy: 40%, 22%; hormone therapy: 23%, 13%).

**Conclusions:**

These results reveal the socio-demographic and clinical heterogeneity of the survivor population, and highlight groups which may have specific medical and supportive care needs. These types of population-based estimates may help decision-makers, planners and service providers to develop follow-up and after-care services to effectively meet survivors’ needs.

## Background

In most countries the number of people diagnosed with cancer is rising steadily. In Europe, an estimated 3.2 million cancers were diagnosed in 2006; by 2012, this had risen to 3.5 million [1, 2]. This is a result of both demographic changes (i.e. population ageing) and changes in underlying risk. At the same time, survival for many cancers is improving – by 1-2% per annum - and 5-year relative survival for all cancers combined now exceeds 50% [3, 4]. These trends mean that there are increasing numbers of “cancer survivors” (i.e. people living with and beyond a diagnosis of cancer [5]) in the population.

In the past, the life trajectory for most people diagnosed with cancer was one of inexorable decline. Nowadays, therapeutic advances, and a greater focus on addressing the side-effects and toxicities of treatment, mean that many people successfully complete primary treatment, recover substantial functional capacity, and can - potentially - resume everyday activities [6]. However, this cannot be taken to suggest that survivors simply return to “normal life”; instead they must find a “new normal” that recognizes and accommodates cancer and its consequences [7–9]. For example, many survivors have complex health conditions which co-exist with their cancer [10], or arise as a consequence of treatment (see, for example, [11–16]). They also have an elevated risk of developing a second cancer [16]. In addition, evidence is accumulating that many survivors experience: significant functional and psychological problems and limitations; social, sexual and relationship difficulties; and financial/economic problems due to cancer and its treatment [17]. Taken together these issues mean that survivors may have considerable ongoing needs for medical and non-medical support and care long after their initial diagnosis and treatment. The prevalence, nature and extent of these supportive care needs vary by survivors’ socio-demographic and clinical characteristics (see, for example, [18–23]). In addition, research from several healthcare systems suggests these needs often go unmet [21].

The resource requirements for supporting cancer survivors are likely to be quite different to those necessary for treating newly diagnosed cancers [24]. Therefore, estimates of the number of people living with cancer in the population - and a description of their characteristics - could aid decision-makers, planners and service providers (both statutory and voluntary, in health and social care) in developing services, supports and other initiatives to meet survivors’ needs.

Population-based cancer registries, which record and follow-up every cancer diagnosed within a defined population, operate in many countries worldwide; in the Europe Union, 26 countries have either regional or national registries [25]. Such registries are considered an essential component of any comprehensive cancer control programme. They provide robust data on cancer incidence, survival, and mortality in the population, and the only truly valid data for: monitoring and projecting the population-level burden of cancer (to inform service planning); assessing variations in incidence (to reveal possible differences in exposure to risk factors and provide information on effectiveness of prevention strategies); and examining patterns and trends in clinical outcomes (to evaluate quality of, and equity of access to, services) [26, 27]. To date, however, registry data has not been extensively used to inform resource requirements for follow-up and after-care services. In order to illustrate how cancer registry data may be useful in survivorship care service planning, we aimed to: (i) generate population-based estimates of cancer prevalence in Ireland; and (ii) describe the socio-demographic and clinical characteristics of the population of people living with cancer. Our secondary aim was to identify subgroups of survivors who might have specific needs in terms of follow-up or after-care services.

## Methods

### Data source

We derived data from the National Cancer Registry (NCR). Since 1994, the NCR has aimed to identify all incident cancers in the population usually resident in Ireland. An active registration process is implemented by trained tumour registration officers (TROs) each of whom has responsibility for recording data on newly diagnosed cancers from a defined group of hospitals. Cases are identified through histopathology reports, the hospital inpatient system, and records from radiotherapy units, oncology wards, day units and pharmacies. Follow-up is achieved by regular linkage with death certificates, provided by the Central Statistics Office, supplemented with information from medical records for a small proportion of cases. Death certificates are matched, using personal identifying details, to cancer registrations using probabilistic linkage methods, supplemented by manual checking. The Registry records date and cause of death for the matched cases. Completeness of registration is estimated to be at least 97% [28].

### Estimating prevalence

We abstracted details of people with all primary invasive cancers (International Classification of Diseases 10^th^ revision (ICD10): C00-C96) diagnosed during 1994–2011, who were still alive on 31/12/2011, thus providing an estimate of 17-year prevalence. We excluded small numbers of cases for which sex (n = 13) or date of birth (n = 340) was unknown. Our initial estimates for all cancers include non-melanoma skin cancers (NMSC; C44) but, since most registries do not record complete data on NMSC, these were excluded from subsequent analyses in order to permit international comparisons. Prevalence is based on individuals rather than tumours so only the first diagnosed tumour for a site was counted. Therefore, in analyses of all cancers combined, people who were diagnosed with multiple primary cancers were counted once, according to the date of diagnosis of their first cancer. The same was done for sites where an individual may have been diagnosed with more than one tumour (e.g. breast). For other sites, an individual with multiple primary cancers contributed to the prevalence for each site (e.g. prostate and lung).

### Statistical analysis

Information on cancer-directed treatments received within a year of diagnosis was used to classify survivors according to whether or not they had received (i) cancer-directed surgery, (ii) chemotherapy, (iii) radiotherapy or (iv) hormone therapy as part of their initial management. Analysis involved categorizing prevalent cancers by cancer site, sex, marital status at diagnosis (married, single, divorced/separated/widowed, unknown), age group at 31/12/2011 (<40, 40–49, 50–59, 60–69, 70–79, ≥80) and time since diagnosis (<1 year, 1–4.99 years, 5–9.99 years, ≥10 years). Associations between the socio-demographic and clinical variables were investigated using chi-square tests.

## Results

In total, 178,813 people in Ireland were diagnosed with an invasive cancer during 1994–2011 and were still alive on 31/12/2011. 90,835 (51%) of these survivors were female and 87,978 (49%) were male. When NMSC was excluded, there were 112,610 cancer survivors at 31/12/2011, of whom 58,054 (52%) were female and 54,556 (48%) were male.

### Cancer site

The most prevalent cancer in both sexes was NMSC: 66,203 people (32,781 females; 33,422 males) were diagnosed with NMSC and no other invasive cancers during 1994–2011 and were still alive at 31/12/2011. Table 1 shows the ten most prevalent cancers in females and males after excluding NMSC. In females, breast cancer (26,066 survivors) dominated with more than four-times as many survivors as the next most prevalent cancer, colorectum (6,598). The third most prevalent cancer was melanoma of the skin (4,593), followed by cancer of the uterus (3,505). Other female gynaecological cancers also featured among the most prevalent sites (cervix, 2,617; ovary, 1,833). In males, prostate cancer was most prevalent (23,966) followed by colorectum (8,207), lymphoma (3,236) and melanoma (2,774).

**Figure 1:**
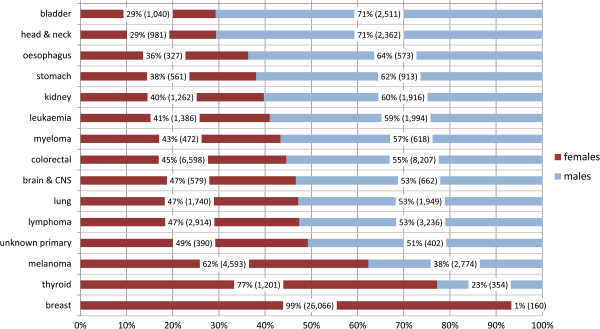
**Gender distribution of survivors, 15 most common sites which affect both sexes: percentages (and numbers).**

When the 15 most common cancers which affect both sexes (other than NMSC) were considered, the proportion of survivors who were female was lowest for bladder and head & neck cancer (both 29%; Figure 1). It was around 40% for cancers of the oesophagus, stomach and kidney and for leukaemia, and almost 50% for cancers of the brain & CNS, lung and unknown primary, and lymphomas. The only sites for which female survivors exceeded males were melanoma (62% female), thyroid (77% female) and breast (99% female).

**Table 1:** **Numbers of people diagnosed with cancer 1994–2011 and still alive at 31/12/2011, by sex and cancer site**

	Females		Males	
***Rank***	***Site (ICD10)***	***No. of people***	***Site (ICD10)***	***No. of people***
1	Breast (C50)	26,066	Prostate (C61)	23,966
2	Colorectal (C18-C21)	6,598	Colorectal (C18-C21)	8,207
3	Melanoma (C43)	4,593	Lymphoma (C81-C85)	3,236
4	Uterus (C54-C55)	3,505	Melanoma (C43)	2,774
5	Lymphoma (C81-C85)	2,914	Bladder (C67)	2,511
6	Cervix (C53)	2,617	Head & neck (C00-C14, C30-C32)	2,362
7	Ovary (C56)	1,833	Testis (C62)	2,257
8	Lung (C33-C34)	1,740	Leukaemia (C91-C95)	1,994
9	Leukaemia (C91-C95)	1,386	Lung (C33-C34)	1,949
10	Kidney (C64-C66)	1,262	Kidney (C64-C66)	1,916

### Age and marital status

**Figure 2:**
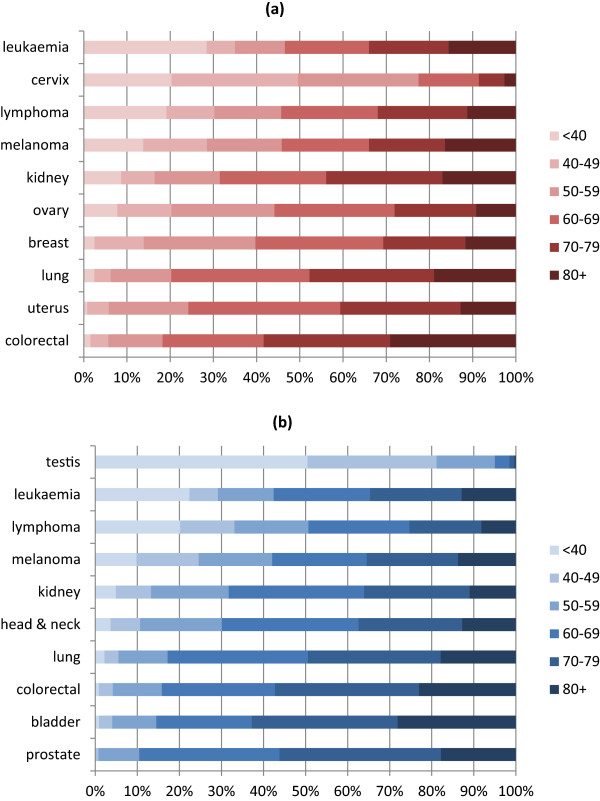
**Percentages of people diagnosed with cancer 1994–2011 and still alive at 31/12/2011, by age at 31/12/2011 and sex, ten most prevalent cancers in females and males (a) Females (b) Males.**

The age distribution of survivors of all invasive cancers (excluding NMSC) differed significantly by sex (Table 2; chi^2^ p < 0.001). Female survivors were younger on average: 39% were aged under 60 at the end of 2011 compared to 25% of males. In contrast, 35% of females were aged 70 or older, compared to 46% of males. The age-distribution of survivors varied by cancer site (Figures 2(a) and (b)). For breast cancer in females, 40% of survivors were under 60, 30% were aged 60–69, 19% were aged 70–79 and 12% were 80 or older. For prostate cancer, 11% were under 60, 33% were 60–69, 38% were 70–79 and 18% were aged 80 or older. Other cancers for which approximately half, or more, of survivors were aged 70 or older were: bladder (males, 63%), colorectum (females, 58%; males, 57%) and lung (females, 48%; males, 49%). The cancers for which there were higher percentages of survivors aged under 40 were: lymphoma (females, 19%; males, 20%), cervix (20%), leukaemia (females, 28%; males, 22%), and testis (50%).

**Table 2:** **Numbers of people diagnosed with cancer 1994–2011 and still alive at 31/12/2011, by sex and age** ^**1**^ **, all invasive cancers** ^**2**^

	Females		Males	
***Age-group***	***No. of people***	***%***	***No. of people***	***%***
<40	4,218	7%	3,644	7%
40-49	6,347	11%	3,097	6%
50-59	12,115	21%	6,984	13%
60-69	15,030	26%	15,513	28%
70-79	11,830	20%	16,479	30%
80+	8,514	15%	8,839	16%
*Total*	*58,054*	*100%*	*54,556*	*100%*

^1^age at 31/12/2011; ^2^excluding non-melanoma skin cancer.

Considering all cancers (excluding NMSC), 61% of survivors were married at the time of diagnosis, 18% were single, 14% were divorced/widowed/separated, and for 7% their marital status was unknown. The marital status distribution varied significantly by sex (p < 0.001): the percentage who were married was lower among females than males (58% vs 65%) the percentage who were single was similar (females 17%; males: 18%); and the percentage who were divorced/widowed/separated was twice as high in females (19% vs 9%).

### Time since diagnosis

**Figure 3:**
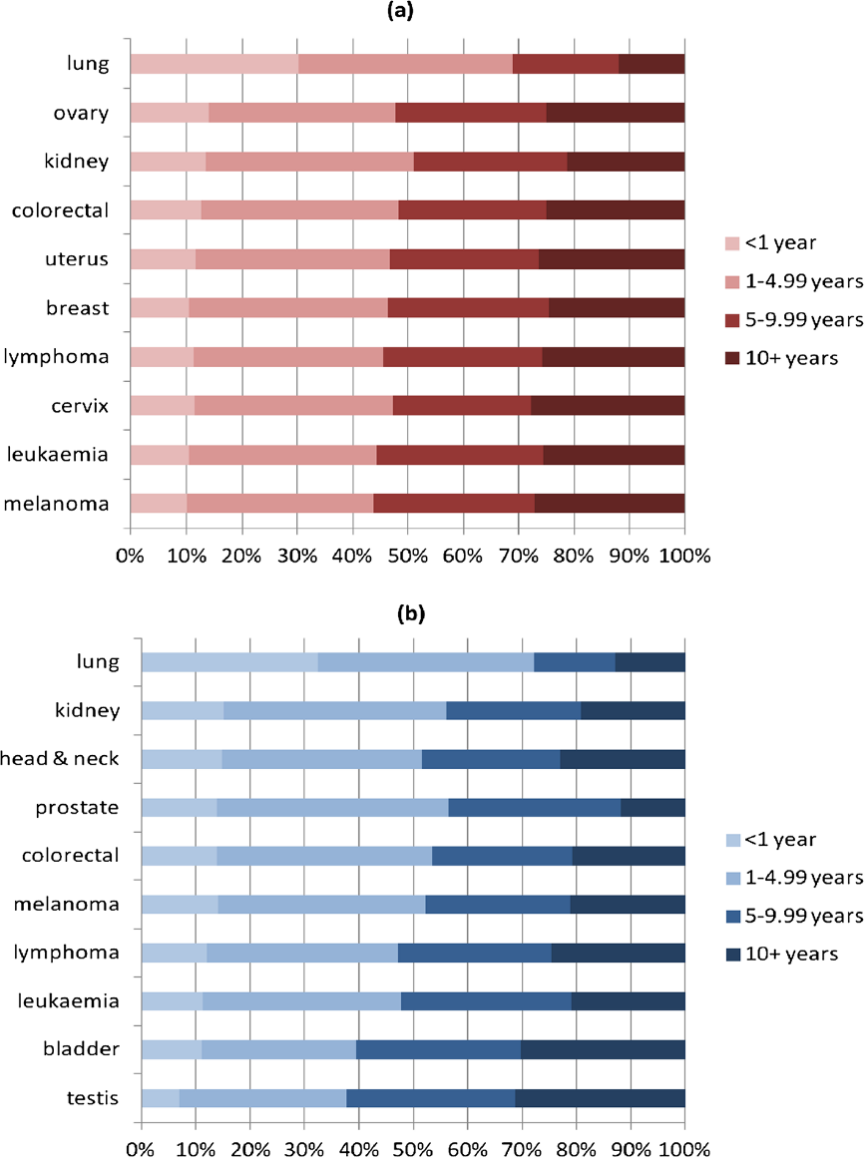
**Percentages of people diagnosed with cancer 1994–2011 and still alive at 31/12/2011, by time since diagnosis** **and sex, ten most prevalent cancers in females and males (a) Females (b) Males.**

For all cancers combined (excluding NMSC), the distribution of time since diagnosis also differed significantly by sex (Table 3; p < 0.001). One quarter of female survivors had lived for 10 years or more with their cancer compared to only 18% of males. For both sexes, for all cancer sites with the exception of lung and testis, 10-15% of survivors had been diagnosed less than one year previously; for lung cancer this figure was 30% in females and 33% in males; for testis cancer, it was 7% (Figure 3(a) and (b)). The percentage of survivors who were at least one year, and less than five years, from diagnosis ranged from 31% (testis) to 43% (prostate). The percentage of long-term survivors (≥10 years) varied between 21% and 28% for all sites in females with the exception of lung (12%). For males, there was more site-specific variation in this percentage: it ranged from 12% and 13% for prostate and lung cancer respectively, to 30% and 31% for bladder and testis cancer, respectively.

**Table 3:** **Numbers of people diagnosed with cancer 1994–2011 and still alive at 31/12/2011, by sex and time since diagnosis** ^**1**^ **, all invasive cancers** ^**2**^

	Females		Males	
***Time since diagnosis***	***No. of people***	***%***	***No. of people***	***%***
<1 year	6,915	12%	7,809	14%
1-4.99 years	20,321	35%	21,156	39%
5-9.99 years	16,224	28%	15,586	29%
10+ years	14,594	25%	10,005	18%
*Total*	*58,054*	*100%*	*54,556*	*100%*

^1^at 31/12/2011; ^2^excluding non-melanoma skin cancer.

### Treatments received

Overall, 69% of survivors had undergone cancer-directed surgery as part of their initial management, 39% had received radiotherapy, 32% had received chemotherapy and 18% had received hormone therapy (Table 4). These percentages were consistently higher for female than male survivors (surgery, 82% vs 54%; radiotherapy, 42% vs 35%; chemotherapy, 40% vs 22%; and hormone therapy, 23% vs 13%). The percentage who had undergone radiotherapy was lowest in the youngest and oldest survivors; chemotherapy receipt decreased with increasing age; and hormone therapy receipt was highest in the oldest age-group. Among the commonest cancers, radiotherapy was most frequent among survivors of prostate (46%), head & neck (65%) and breast (66%) cancer. More than half of survivors with breast cancer (51%), leukaemia (56%), ovarian cancer (64%) and lymphoma (74%) had received chemotherapy. Half of breast cancer survivors and 28% of prostate cancer survivors had received hormone therapy.

**Table 4:** **Number and percentage of people diagnosed with cancer 1994–2011 and still alive at 31/12/2011, who had had cancer-directed surgery, chemotherapy, radiotherapy or homone therapy, overall and by age and time since diagnosis** ^**1**^ **, all invasive cancers** ^**2**^

	Cancer-directed surgery		Chemotherapy		Radiotherapy		Homone therapy	
	***No.***	***%***	***No.***	***%***	***No.***	***%***	***No.***	***%***
*Age-group*								
<40	5,113	65%	3,915	50%	2,147	27%	302	4%
40-49	7,630	81%	4,330	46%	3,760	40%	1,369	14%
50-59	14,855	78%	8,278	43%	8,471	44%	3,693	19%
60-69	21,226	69%	9,950	33%	13,284	43%	5,603	18%
70-79	17,801	63%	6,921	24%	11,658	41%	5,669	20%
80+	10,831	62%	2,315	13%	4,070	23%	3,706	21%
*Time since diagnosis*								
<1 year	7,422	50%	3,350	23%	2,970	20%	1,470	10%
1-4.99 years	27,804	67%	13,831	33%	17,746	43%	8,457	20%
5-9.99 years	22,796	72%	10,553	33%	13,789	43%	6,283	20%
10+ years	19,434	79%	7,975	32%	8,885	36%	4,132	17%
*Total*	*77,456*	*69%*	*35,709*	*32%*	*43,390*	*39%*	*20,342*	*18%*

^1^at 31/12/2011; ^2^excluding non-melanoma skin cancer.

## Discussion

Prevalence is increasingly considered an important measure of the population-level cancer burden. Although some recent studies have estimated prevalence (see [29] and references therein), few (if any) set out explicitly to examine socio-demographic and clinical heterogeneity within the survivor population in order to inform the development of strategies for service provision around cancer follow-up and supportive care.

We estimated that, in total, 178,813 people had been diagnosed with an invasive primary cancer in Ireland during 1994–2011 and were alive at the end of 2011. Expressed crudely in relation to the 2011 population, this means that at least 3.9% of the population of Ireland are cancer survivors. In addition, it implies that each general practitioner has, on average, 60 patients who are cancer survivors. The NCR is unusual in that it aims to record all NMSC cases. Since most other registries do not, in order to permit international comparisons, we repeated our analysis excluding NMSC. When this was done, there were 112,610 survivors, representing 2.4% of the male, and 2.5% of the female, population. These figures are very close to the estimate of 18-year prevalence at the end of 2010 in Northern Ireland (2.5%) [30]. In contrast they are somewhat lower than estimates for Switzerland, Italy and the United Kingdom (UK) [31–33], but these studies estimated complete prevalence (i.e. all survivors) whereas we considered limited duration (i.e. 17-year) prevalence because national cancer registration was not established until 1994. Therefore, the figures in this paper provide a lower bound for the total survivor population in Ireland, and thus the number of people who may require some level of access to cancer follow-up and/or after-care services.

Because prevalence is a function of incidence and survival, there are some notable differences between the cancers which rank highest when incidence or mortality are considered and those which are most prevalent. For example, testicular cancer, which is relatively uncommon but has good survival (175 new cases and 5 deaths per annum in Ireland [4]), is the 14^th^ most commonly-diagnosed cancer among men but ranked 7^th^ in terms of prevalence. In contrast, lung cancer - the 3^rd^ most commonly diagnosed cancer and most common cause of cancer death in both sexes [4] - ranked 8^th^ (females) and 9^th^ (males) when prevalence was considered. Similar patterns are evident in Northern Ireland [30] and the United States [34]. The implication of these observations for service providers is that the cancer-specific composition (or configuration) of services for diagnosis and treatment should be different from that for rehabilitation, follow-up and after-care services (because the composition of the types of cancers requiring diagnosis/treatment and follow-up/after-care differs).

The rationale for examining prevalence by time since diagnosis is that survivors at different “phases” of follow-up/survivorship may have different needs. For example, many of those who have survived the first year post-diagnosis may be in need of (or benefit from) rehabilitation services [35]; the higher prevalence of anxiety in those who have survived two or more years post-diagnosis than among controls [36], suggests psychological support services could be of benefit to notable proportions of intermediate-term survivors; while long-term survivors (i.e. survived ≥10 years) may be in need of services focused on the detection and management of late-effects of treatment and/or second primaries. The distribution of time since diagnosis for all invasive cancers in this analysis was almost identical to that seen in Northern Ireland [30]. The observed higher percentage of longer-term survivors among females is driven in large-part by breast cancer: almost 6,500 women had survived for ≥10 years after a breast cancer diagnosis. Moreover, two-thirds of breast cancer survivors had received radiotherapy and half had received chemotherapy. Radiotherapy to the breast and trastuzamab are associated with risk of late cardiac complications [11, 12] and those responsible for follow-up care for long-term breast cancer survivors need to be alert to these risks.

We found that 15% of survivors were younger than 50 and a further 17% were aged 50–59. The concerns, and hence burden of supportive care needs, of younger and older survivors may be quite different [23]. For example, younger survivors may be more concerned about employment and related financial matters, relationships, and fertility and sexuality issues [37–39]. In addition, they may adapt less well to having cancer than older survivors [40]. Our findings with regard to the age distribution of different cancers suggest that follow-up and after-care services and supports for survivors of leukaemia, lymphoma and melanoma and testicular and cervical cancer should encompass these types of issues.

At the other end of the age spectrum, four in every 10 survivors were aged 70 or older. This means that at least 12.6% of this age-group in Ireland (16.1% of males; 9.9% of females) has a history of invasive cancer other than NMSC. Older survivors tend to have more comorbidities than younger survivors [40]. They also have high levels of psychological distress related to the continuing effects of cancer and its treatment, significant limitations in physical functioning, and higher rates of frailty than the general population [41–43]. These issues are inter-related and may be exacerbated by lifestyle factors [42, 44]. Our results therefore suggest that follow-up and after-care services for survivors of bladder, colorectal and lung cancer, in particular, should be linked closely to geriatric and other non-cancer specialties, and should be broad ranging. It is noteworthy, however, that while there have been trials of alternative models of follow-up for some of these cancers [45, 46], models specifically focused on older survivors needs, or which explicitly seek to involve geriatric and other-specialties, do not appear to have been evaluated.

In some studies unmarried survivors have higher supportive care needs [23]. Thirty-two percent of our survivor population was not married at the time of diagnosis, and this figure was higher among older survivors (≥70, 35%; ≥80, 47%), providing further evidence to suggest that the elderly survivor population may have pronounced needs. In Ireland, among the elderly, living alone is a marker of poverty and experiencing multiple types of enforced deprivation [47]. Since socio-economic status influences access to cancer care [48–51], in developing supportive care services it will be important to consider strategies and approaches for minimizing inequalities in access.

The higher proportion of female to male survivors in this study echoes findings elsewhere [30, 34] and is a function of the dominance of breast cancer among women. As in the United States and UK [33, 34], more than 40% of female survivors in Ireland had a history of breast cancer. Among breast cancer survivors unmet needs are common, are present across the survivorship continuum, and often relate to emotional or existential concerns (see, for example, [52, 53]), suggesting that after-care services for both shorter-term and longer-term breast cancer survivors should encompass these types of issues.

Just under half of survivors in Ireland were male (48%), higher than figures from Northern Ireland (43%) and the UK as a whole (41%) [30, 33]. This is due to prostate cancer incidence in Ireland, which was estimated to be the highest in Europe in 2008 [54]. This high incidence is a consequence of widespread prostate specific antigen testing in primary care [55]. Indeed, 42% of the male survivors in Ireland had been diagnosed with prostate cancer, almost identical to the US (43%) [34]. Prostate cancer treatments are associated with significant side-effects which can impact adversely on health-related quality-of-life [56]. Prevalence of related side-effects is high, even years after treatment [57] and, although there are interventions available for the management of these, men often do not receive information about what is available (M Hennessy, personal communication). This suggests that there may be significant needs for physical, psychological and psychosexual support among prostate cancer survivors.

This study was based on high-quality cancer registration data. Although registration completeness is high [28], a small proportion of cases is missed by the Registry, meaning that these figures slightly under-estimate true 17-year prevalence; the extent of this under-estimation is likely to vary by site. Accurate prevalence estimates also require comprehensive death registration and the ability to perform accurate linkage between death certificates and cancer registrations. While death ascertainment is likely to be high in Ireland, it is possible that some people diagnosed with cancer left Ireland and subsequently died before the end of 2011; these deaths would not be known to the Registry. As noted earlier, these figures do not claim to be estimates of the total number of survivors – in particular, they underestimate the true number of long-term survivors (i.e. they do not include people diagnosed with cancer before 1994 and who were still alive at the end of 2011). While methods are available for estimating total prevalence [58, 59], these require assumptions which may not be valid. Moreover, it might be argued that many of those who have survived cancer for at least 18 years are likely to be at low risk of recurrence and have little need for active follow-up. Therefore, it has been suggested that limited duration prevalence is likely to be more pertinent for estimating the needs for cancer services according to specific phases of cancer care [29]. In terms of other limitations, these analyses do not identify those survivors whose cancer was cured, those in active therapy or those dying from cancer; this information is not available through the Registry. Nor do they reveal anything directly about the health status, or unmet supportive care needs, of survivors. Evidence is accruing that aspects of health-related quality-of–life may vary by survivors’ socio-economic, urban/rural, or immigrant status [60–64] suggesting that supportive care needs may also vary and, in turn, that estimates of the prevalence of survivors in different socio-economic groups, for example, could be valuable for service planning. We did not consider these characteristics and this is a limitation of the study. Finally, these figures are a snap-shot of prevalence at one point in time. As in other developed countries, cancer incidence in Ireland will continue to rise in coming years [65]; even if survival does not improve, the numbers of cancer survivors will grow, particularly in the older age groups. Estimates of this future burden are also needed to help providers make provision for this booming population.

## Conclusion

In conclusion, this study shows that data from cancer registries can provide a population-based estimate of the number of cancer survivors, information likely to be of considerable value to service planners and providers in the statutory and voluntary sectors. They also reveal important heterogeneity within the survivor population - which is likely to determine (at least in part) their ongoing medical and supportive care needs and hence influence service requirements - and provide an indication of the likely magnitude of groups of survivors who may have specific service and support needs (e.g. testicular cancer survivors, survivors of leukaemia and lymphoma, long-term breast cancer survivors, unmarried survivors, elderly survivors). Figures such as these provide an important first step in informing development of follow-up and after-care services, supports and other initiatives which will effectively meet survivors’ needs.

## Abbreviations

ICD10: International Classification of Diseases 10^th^ revision
NCR: National Cancer Registry
NMSC: Non-melanoma skin cancer
TRO: Tumour registration officer
UK: United Kingdom.
